# Determinants of government spending on primary healthcare: a global data analysis

**DOI:** 10.1136/bmjgh-2023-012562

**Published:** 2023-11-30

**Authors:** Darius Erlangga, Timothy Powell-Jackson, Dina Balabanova, Kara Hanson

**Affiliations:** Department of Global Health and Development, London School of Hygiene & Tropical Medicine, London, UK

**Keywords:** Health systems, Health economics

## Abstract

In 2018 global leaders renewed their political commitment to primary healthcare (PHC) ratifying the Declaration of Astana emphasising the importance of building a sustainable PHC system based on accessible and affordable delivery models strengthened by community empowerment. Yet, PHC often remains underfunded, of poor quality, unreliable and not accountable to users which further deprives PHC of funding. This paper analyses the determinants of PHC expenditure in 102 countries, and quantitatively tests the influence of a set of economic, social and political determinants of government expenditure on PHC. The analysis is focused on the determinants of PHC funding from government sources as the government is in a position to make decisions in relation to this expenditure as opposed to out-of-pocket spending which is not in their direct control. Multivariate regression analysis was done to determine statistically significant predictors.

Our analysis found that some economic factors—namely Gross Domestic Product (GDP) per capita, government commitment to health and tax revenue raising capacity—were strongly associated with per capita government spending on PHC. We also found that control of corruption was strongly associated with the level of total spending on PHC, while voice and accountability were positively associated with greater government commitment to PHC as measured by government spending on PHC as a share of total government health spending.

Our analysis takes a step towards understanding of the drivers of PHC expenditure beyond the level of national income. Some of these drivers may be beyond the remit of health policy decision makers and relate to broader governance arrangements and political forces in societies. Thus, efforts to prioritise PHC in the health agenda and increase PHC expenditure should recognise the constraints within the political landscapes and engage with a wide range of actors who influence decisions affecting the health sector.

**What is already known on this topic**

- Given the lack of comparable data on primary healthcare (PHC) expenditure over time, only a few studies have explored the determinants of PHC expenditure at the global level.
- This literature is mainly limited to high-income countries and tends to focus on specific elements of PHC expenditure such as outpatient care or pharmaceuticals.

**What this study adds**

- While it is expected that richer countries spend more money on PHC per capita, we found no evidence that they also allocate more (or less) of the government health budget to PHC.
- Holding income constant, a country’s capacity to raise tax revenue was found to be an important determinant of the level of government spending on PHC.
- None of the social variables were significant predictors of government spending on PHC.
- Political factors may exert some influence on the level of spending and the prioritisation of PHC.

**How this study might affect research, practice or policy**

- This study provides some preliminary insight on how to increase prioritisation of financing PHC at the global level.
- While economic factors are still the important determinants, recognising the political landscape of the PHC system is critical to increase allocation of government budget to PHC.

## Background

The 1978 Alma Ata Declaration defined primary healthcare (PHC) as a cornerstone of an effective health system and recognised it as a global priority. Following decades in which this vision of PHC proved difficult to sustain, in 2018 global leaders renewed their political commitment to PHC ratifying the Declaration of Astana which emphasised the importance of building sustainable PHC by providing accessible and affordable delivery models and empowering individuals and communities to demand and participate in their healthcare.^1^

Adequate investment in PHC should enable health systems to reduce the need for unnecessary hospital admissions, prevent avoidable readmissions and limit inappropriate use of emergency departments.^2^ Modelled estimates show that increasing PHC expenditure from an average 5.6% of GDP to 6.6% of GDP in 2030 in 67 low-income and middle-income countries (LMICs) could avert up to 64 million deaths.^3^ Yet despite these advantages, PHC often remains underfunded, of poor quality, unreliable and not accountable to users. In many LMICs, users bypass PHC facilities, instead seeking higher-level specialist care and incurring considerable costs in the process.^4 5^ This cycle further deprives PHC of funding, and the lack of resources further exacerbates the problems that have driven patients away from PHC facilities. To break this cycle, countries need to prove their commitment to PHC by allocating more resources to PHC while also striving for more efficient use of the existing resources.

To understand how much more funding is needed for PHC it is first necessary to measure what is currently spent. Empirical spending data allow policy makers to track existing expenditure, demonstrate how funds are currently being used, and make a case for increased commitments. The first comparable national estimates of PHC spending were generated in 2017 as part of the Global Health Expenditure Database maintained by WHO. Once the current level of PHC spending is known, it is critical to understand the factors that influence how much countries spend on PHC. Countries could learn from others about the optimal conditions that are likely to support their policies in increasing resources to PHC.

Given the lack of comparable data on PHC expenditure over time, only a few studies have explored the determinants of PHC expenditure at the global level. This literature is mainly limited to high-income countries,^6–8^ and tends to focus on specific elements of PHC expenditure such as outpatient care or pharmaceuticals.^9 10^ In LMICs, donor funding typically covers vertical programmes delivered at PHC level, however increased domestic resources play an important role to sustain PHC expenditure.^11^ PHC expenditure may be higher in settings with higher morbidity rates (eg, non-communicable disease risk factors^6 8^) and those with higher availability of health workers (eg, the number of family doctors^7 12^). Yet these studies do not address the determinants of PHC expenditure at the health-system level.

There is emerging evidence that the level of PHC expenditure may be influenced by forces in the wider political context. The 2022 Lancet Global Health Commission on Financing PHC argued that a country’s decision to prioritise PHC within their health spending and create mechanisms to channel it to PHC services is not only technical.^13^ An examination of country experience drawn from case studies and other qualitative evidence suggests that PHC expenditure is frequently shaped by political, social and economic factors, often interrelated and acting together.

However, in the absence of data to examine these relationships for PHC expenditure, most empirical studies of these variables have analysed their relationship with total health expenditure rather than PHC expenditure. These have found determinants of overall health expenditure to include not only macroeconomic factors (eg, GDP per capita as the strongest determinant),^14–18^ health financing arrangements,^19 20^ and demographic and socioeconomic status,^21 22^ but critically, political factors.^23–28^ Reeves *et al* found the implementation of austerity measures in 27 European Union (EU) countries led to reduced health expenditure, but that the government’s ideology had no impact.^23^ Both democratic and authoritarian leadership have been shown to have a positive effect on government health expenditure (GHE).^24–26^ Decentralisation may be important, with studies showing that countries with highly decentralised political structures have higher health expenditure, possibly arising from a tendency to respond to population demands for public services expressed via local election cycles.^25 29^ In India, states with more intense political competition, higher degree of decentralisation and higher level of electoral turn-out had higher government healthcare spending across.^27^ Democratic accountability was found to have a diminishing positive correlation with GHE, however the levels of GHE were higher when the government was more stable.^28^ Corruption is associated with lower GHE in LMICs, but with higher GHE in high-income countries.^28^

Political will and good governance in health have been shown to be major drivers of effective health systems^30^—often achieved through strengthening PHC and improving service accessibility and equity, and particularly among women and children. Particular governance arrangements and political structures have been associated with improved health outcomes, such as maternal, child and adult mortality and life expectancy, intermediated by the strength of the health system and the level of resources.^31 32^ However, there is limited empirical work on the political determinants of PHC spending, for example, the association between governance models (nature or effectiveness of democracy, etc) and investing in PHC, measured by the level and share of PHC spending in total government spending.

A plausible proposition is that the political factors such as better accountability in society and efforts to direct resources to those in most need such as the poorest and most vulnerable groups who are most likely to use PHC, may promote increased spending on PHC. This is enshrined in the Sustainable Development Goals (SDGs) such as SDG 16 that envisages that effective public institutions including health systems should be underpinned by social justice. Achieving this goal may require prioritisation of PHC within government budgets, increasing the overall level of spending for PHC. Better transparency, accountability and control of corruption may be associated with higher spending across the board, including PHC, as globally it is estimated that at least 10%–25% of healthcare funding is lost to corruption annually.^33^ Evidence from China and India suggests that democratic pressure from citizens may increase public spending more broadly.^34^

This paper examines the determinants of PHC expenditure using a cross section of countries, with a particular focus on the determinants of PHC funding from government sources as the government is in a position to make decisions in relation to this expenditure as opposed to out-of-pocket spending which is not in their direct control. We do this by analysing the determinants of PHC expenditure in 102 countries, and quantitatively testing the influence of a set of economic, social and political determinants of expenditure on PHC.

## Methods

### Approaches to measuring PHC expenditure

Analysing the financing arrangements for PHC requires clarity about what is included in measures of PHC expenditure. Measuring PHC spending is challenging, primarily because PHC can include functions outside what is typically considered the health sector and, even within the health sector, there is no clear consensus on how to operationalise the definition of PHC. The System of Health Accounts (SHA)^35^ provides the standard framework for measuring health expenditure but it does not directly track expenditure on PHC services. Instead, proxy measures derived from the SHA must be used, the most prominent of which were developed by the Organisation for Economic Co-operation and Development (OECD) and WHO, respectively.

The OECD first developed a measure of PHC expenditure using a definition based on the cross-classification of healthcare functions (including general outpatient curative care, outpatient dental care, home-based curative care and preventive care) and healthcare providers (ambulatory providers).^36^ WHO subsequently proposed a broader approach to include 80% of total medical goods provided outside health facilities.^37^ It also assigned 80% of total expenditure on governance and administration to PHC. Other approaches have been described in the literature. For instance, Maele *et al* explored eight operational options to measuring PHC with a focus on LMICs but did not propose a new indicator.^38^ In another effort to define PHC for the purpose of expenditure tracking, Baillieu *et al* proposed a tiered framework that is based on first contact, continuity, comprehensiveness and coordination, but at this time there is no database that provides indicators supporting this framework.^39^

Both the OECD and WHO have databases containing estimates of PHC expenditure based on their preferred definition, with some overlap in the countries covered. The OECD database covers 32 OECD (high-income) countries and produces estimates of total PHC expenditure and government PHC expenditure (based on financing scheme). The WHO database covers 102 countries, of which 32 are OECD countries. It disaggregates PHC expenditure by source of financing only for the non-OECD countries. To enable the widest coverage of countries for this analysis we opted to use the WHO definition of PHC expenditure, with one important modification: as is done in the OECD estimates, we excluded expenditure on governance and health system administration, on the grounds that it is both unrealistically high (and without clear justification or explanation for its 80% share assigned to PHC), and that it heavily distorts expenditure on PHC in low-income countries, with governance and administration accounting for an average of 42% of total government spending on PHC in low-income countries.

### Data sources

Our main source of data was the WHO Global Health Expenditure Database. It reports data on total PHC spending for 104 countries in 2018 and 97 countries in 2019. This database also reports government spending on PHC for 69 out of 104 countries in 2018 and 64 out of 97 countries in 2019. The WHO database only provides estimates of total (not government) PHC spending for OECD countries. In order to include OECD countries in our analysis we used the OECD database to compile *approximate* estimates of government expenditure manually for the 32 OECD countries using the WHO PHC definition. This approximation method was to estimate the missing government spending for PHC in 32 OECD countries because the WHO database already reported total PHC spending for these countries. It should be noted that while it was possible to manually compile approximate expenditure estimates for OECD countries using the WHO definition, it was not possible to produce estimates for countries in the WHO database using the OECD definition of PHC expenditure that includes services provided by ambulatory providers only.

We used information in the WHO database on the exchange rate and size of population to convert the estimates of PHC expenditure from local currency to current 2019 US$ values. For countries with missing data in 2019, we used expenditure reported in 2018 or earlier, if available, which increased our sample size from 97 to 113 countries (see online supplemental appendix 1 for more detail). However, we needed to exclude 11 countries (see online supplemental appendix 1) out of those 113 countries because of missing data on explanatory variables. Our final sample size was 102 countries. Throughout the analysis, we used explanatory variables from year 2019.

10.1136/bmjgh-2023-012562.supp1 Supplementary data

### Outcome variables

The main outcome used in the analysis was government PHC spending per capita as this is the measure of expenditure most amenable to the influence of national authorities, which is our primary concern. Government spending on PHC excludes any donor funding disbursed through the government budget. We also analysed two additional measures of expenditure: total PHC spending per capita and government spending on PHC as a share of government health spending. The former includes private spending and external aid on PHC and is therefore a holistic measure of PHC expenditure. The latter is a measure of the government’s commitment to PHC.

### Predictors

To guide our analysis, we used the political economy framework adopted in the Lancet Global Health Commission on PHC financing,^13^ that explicitly considered three major set of factors that influence government financing for PHC. The first area was economic conditions which are hypothesised to influence PHC spending by determining the overall resource envelope available for health spending and for PHC expenditure. We considered variables measuring various aspects of the economy, including GDP per capita, tax revenue as a share of GDP and government health spending as a share of general government spending. These variables were taken from the World Bank’s World Development Indicators.^40^

The second domain was social conditions, encompassing social and cultural values, informal networks, class, caste or other social constructs that can influence allocation of resources. We explored the following variables that represent this domain and which were shown in existing studies to be associated with health system performance. From the World Development Indicators^40^ we obtained information on age structure, proportion of the population living in urban areas, population density and Gini coefficient (a common measure of income inequality). High proportion of either youth (<15) or elderly (>65) population has been associated with higher health expenditure.^21^ More urbanised countries^41^ and high population density^42^ are associated with higher health expenditure. Cultural diversity has been shown to influence the distribution of health utilisation and health outcomes.^32 43^ To measure cultural diversity, we used the Ethnic Fractionalisation Index data set.^44^ Social capital was also suggested as an important factor within the social conditions domain as increased social capital may have a positive impact on health,^45^ and we sourced a quantitative measure of social capital from The Legatum Prosperity Index.^46^ Lastly, gender inequality, which has been shown to be associated with health outcomes,^47 48^ was hypothesised to also influence allocations to PHC as women are more likely to use PHC which is often seen as the lower priority segment of the health system. This was included through the Gender Inequality Index.^49^

The third domain was politics, including the range of political actors, their relationships and contracts, their legitimacy, as well as contestation or collaboration leading to the enactment of policies. Choosing the appropriate data sets to measure these factors proved to be challenging due to the mixed results on associations with outcome indicators reported in the literature and also to the limited coverage of countries in some data sets. Finally, we settled on the following variables to represent political forces in our analysis. We used six governance indicators from Worldwide Governance Indicators (see table 1).^50^ We also used other databases that provide information about political economy variables relevant to health: the Government Closeness Index as a proxy for measuring decentralisation,^51^ the degree of autocracy and democracy from the Polity IV project database,^52^ and the Liberal Democracy Index from the Varieties of Democracy (V-Dem) data set.^53^ Table 1 lists all of the predictor variables with some description and the data source.

**Table 1:** Explanatory variables considered in the analysis

Variable name	Description	Source
Economic factors		
GDP per capita		World Development Indicators^40^
Tax revenue as % of GDP	Tax revenue including social contribution.	International Monetary Fund (IMF)^58^
Government commitment to health	Government health spending as a share of general government spending.	World Development Indicators^40^
Social factors		
Age structure	Proportion of population aged >65 years old and <15years old.	World Development Indicators^40^
Urban/rural	Proportion of population living in urban area.	World Development Indicators^40^
GINI coefficient	Income inequality.	World Development Indicators^40^
Ethnic Fractionalisation Index	It corresponds to the probability that two randomly drawn individuals within a country are not from the same ethnic group, to show the pattern of ethnic diversity across countries.	Drazanova 2019 44
Social capital	It measures how cohesive a society is in terms of people trusting, respecting and helping one another, and the institutional structures they interact with.	Legatum Prosperity Index^46^
Gender Inequality Index	It measures gender inequalities in three important aspects of human development—reproductive health, empowerment and labour market participation.	United Nations Development Programme (UNDP)^49^
Political factors		
Voice and accountability	Perceptions of the extent to which a country’s citizens are able to participate in selecting their government, as well as freedom of expression, freedom of association and a free media.	World Governance Indicators^40^
Political Stability	Perceptions of the likelihood of political instability and/or politically motivated violence, including terrorism.	World Governance Indicators^40^
Government effectiveness	Perceptions of the quality of public services, the quality of the civil service and the degree of its independence from political pressures, the quality of policy formulation and implementation, and the credibility of the government’s commitment to such policies.	World Governance Indicators^40^
Regulatory quality	Perceptions of the ability of the government to formulate and implement sound policies and regulations that permit and promote private sector development.	World Governance Indicators^40^
Rule of law	Perceptions of the extent to which agents have confidence in and abide by the rules of society, and in particular the quality of contract enforcement, property rights, the police and the courts, as well as the likelihood of crime and violence.	World Governance Indicators^40^
Control of corruption	Perceptions of the extent to which public power is exercised for private gain, including both petty and grand forms of corruption, as well as ‘capture’ of the state by elites and private interests.	World Governance Indicators^40^
The government closeness index/decentralisation	It measures government decision making at the local level, that is, the level of government closest to the people. It captures institutional dimensions of political, fiscal and administrative autonomy enjoyed by local governments.	Ivanyna and Shah^51^
POLITY Score	It is computed by subtracting the AUTOCRATIC Score from the DEMOCRATIC Score; the resulting unified polity scale ranges from +10 (strongly democratic) to −10 (strongly autocratic).	POLITY5 project^52^
Liberal Democracy Index	The liberal principle of democracy emphasises the importance of protecting individual and minority rights against the tyranny of the state and the tyranny of the majority.	V-DEM data set^53^

PHC, primary healthcare.

### Statistical analysis

From the existing literature on the determinants of total health expenditure, GDP is expected to be a major determinant of both the level of government spending on PHC and the PHC share of government health spending. As the distributions of both health expenditure and GDP are typically highly skewed to the right we used a logarithmic transformation to aid in interpretation and improve model fit.^54^ A series of bivariate Ordinary Least Squares (OLS) regressions of the health expenditure variables over each independent variable were performed to provide a baseline against which we could compare the results from the next stage. In a second stage, multivariate regression equations were estimated to assess these associations simultaneously while controlling for GDP and all other predictors. At this stage, we excluded some predictors from the final model due to their non-significant results and because missing values for some variables led to a material reduction (18 countries) in the analytical data set. The results of keeping all predictors in the model are shown in the appendix as a comparison. We also conducted a test of multicollinearity by calculating the variance inflation factor (VIF) for each predictor. VIFs exceeding 4 warrant further investigation while VIFs exceeding 10 are signs of serious multicollinearity. All statistical analysis was performed using Stata SE V.17.

### Patient and public involvement

This study is based on publicly available aggregate data at the country level. No patients or members of the public were involved in the design of this study

## Results

Table 2 presents summary statistics for outcomes and independent variables in the countries for which data on government spending on PHC per capita were available. Total PHC expenditure per capita ranges from $10.55 per capita (Democratic Republic of the Congo - DRC) to $3520 per capita (Switzerland). The same pattern can be seen for government spending on PHC per capita, which ranges from a mere $1 per capita (DRC) to $2192 per capita (Norway). The mean total PHC expenditure per capita was $464 across the 102 countries in the sample, while the mean government PHC expenditure was $286 per capita. There was no correlation between the level of government spending on PHC and the share of government spending on PHC out of total government health spending (correlation coefficient 0.09 with value of p=0.35). For example, DRC had the lowest level of per capita government spending on PHC at $1, but its share of PHC in total government health spending was 31%. In contrast, Norway had the highest level of government spending on PHC per capita, but only spent 32% of its health budget on PHC.

**Table 2:** Summary statistics for outcome and independent variables (2019 or latest figure)

Variable	Year	N	Mean	SD	Min	Max
Outcome						
Total spending on PHC per capita (US$)	Latest	102	464.13	757.39	10.55	3520.16
Government spending on PHC per capita (US$)	Latest	102	285.77	520.11	1.01	2192.57
Government spending on PHC as a share of total government health spending	Latest	102	35.15	13.01	7.72	72.78
Economic factors						
GDP per capita (US$)	2019	102	15 048.11	22 155.85	374.67	115 826.10
Government health spending as % of government spending	2019	102	10.02	4.73	2.11	24.21
Tax revenue including social contribution as % of GDP	2019	102	21.10	10.69	0.00	46.60
Social factors						
% of young population (<15 years)	2019	102	62.73	6.53	47.56	84.88
% of population >65 years	2019	102	8.88	6.63	1.16	28.00
% Urban population	2019	102	55.63	22.77	16.52	99.19
Gini coefficient (latest figure)	Latest	102	37.86	10.42	24.60	99.70
Ethnic Fractionalisation Index	2013	90	0.49	0.26	0.02	0.89
Population density (population per square kilometre area)	2019	102	126.57	130.24	2.97	623.30
Gender Inequality Index	2021	95	0.35	0.20	0.03	0.68
Social Capital Index	2019	96	53.40	10.53	22.50	81.42
Political factors						
Control of corruption	2019	102	0.04	1.01	−1.77	2.16
Rule of law	2019	102	0.05	0.99	−1.97	2.06
Regulatory quality	2019	102	0.05	0.95	−2.05	1.87
Government effectiveness	2019	102	0.04	1.01	−2.45	2.01
Political stability and absence of violence/terrorism	2019	102	−0.07	0.92	−2.66	1.64
Voice and accountability	2019	102	0.00	0.95	−1.99	1.66
POLITY regime measure	2018	96	4.72	5.80	−10.00	10.00
Government Closeness/Decentralisation Index	2012	98	2.63	5.36	0.00	34.03
Liberal Democracy Index	2019	99	0.44	0.26	0.05	0.89

PHC, primary healthcare.

Table 3 reports the results from bivariate regressions for all three outcome variables. For the total PHC expenditure per capita (column 1) and government PHC expenditure per capita (column 2), all predictors showed a significant correlation except for the Gini coefficient and population density. For government PHC expenditure as a share of total GHE (column 3), none of the variables showed a statistically significant correlation.

**Table 3:** Results of bivariate regression for all predictors and outcomes

Predictors	N	(Log) per capita total PHC spending	(Log) Per capita government PHC spending	Government PHC spending as a share of total government health spending
		(**I**)	(**II**)	(**III**)
GDP per capita (US$) in log format	102	1.00**	1.38**	−0.34
Government health spending as % of government spending	102	0.25**	0.36**	−0.25
Tax revenue including social contribution as % of GDP	102	0.12**	0.17**	0
% of young population (<15 years)	102	−0.07**	−0.11**	0
% of elderly population (>65 years)	102	0.17**	0.22**	−0.14
% Urban population	102	0.05**	0.07**	−0.04
Gini coefficient (latest figure)	102	−0.03	−0.05	0.11
Ethnic Fractionalisation Index	90	−0.03**	−0.04**	0.03
Population density (population per square kilometre area)	102	0	0	0
Gender Inequality Index	95	−0.07**	−0.09**	0.03
Social Capital Index	96	0.10**	0.14**	−0.06
Control of corruption	102	1.35**	1.88**	0.73
Rule of law	102	1.41**	1.96**	−0.39
Regulatory quality	102	1.50**	2.03**	−0.73
Government effectiveness	102	1.39**	1.95**	−0.27
Political stability and absence of violence/terrorism	102	1.24**	1.83**	0.18
Voice and accountability	102	1.24**	1.72**	1.48
POLITY regime measure	96	0.11**	0.15**	0.22
Government Closeness/Decentralisation Index	98	0.18**	0.24**	0.16
Liberal Democracy Index	99	0.05**	0.06**	0.04

*p<0.05, **p<0.01.

PHC, primary healthcare.

Table 4 reports the results from multivariate regressions for two outcomes: the log of government spending on PHC per capita and the log of total spending on PHC per capita. In this table, we excluded Ethnic Fractionalisation Index, Gender Inequality Index, social capital, POLITY Score, Decentralisation Index and Liberal Democracy Index as these were all shown not significant in the full regression and had missing values. When we retained all predictors with missing values our sample size dropped from 102 to 84, and we elected to prioritise keeping our sample size intact. The results of regressions with all predictors is shown in the appendix (see online supplemental appendix 2).

**Table 4:** Results of multivariate regression for the level of government spending on PHC (2019 or latest figure)

Predictors	Outcome: Log(Government spending on PHC per capita) (1)			Outcome: Log(Total spending on PHC per capita) (2)		
	Effect size	95% CI	p-value	Effect size	95% CI	P value
Government health spending as % of general government spending †	0.07*	(0.01,0.13)	0.02	0.02	(−0.02,0.05)	0.31
Ln(GDP per capita)	1.01**	(0.82,1.21)	<0.01	0.84**	(0.67,1.01)1	<0.01
Tax revenue including social contribution as % of GDP†	0.04**	(0.01,0.06)	0.01	0.02*	(0.00,0.04)	0.04
% of young people (<15 years old)†	−0.02	(−0.00,0.04)	0.11	<0.01	(−0.02,0.02)	0.89
% of elderly (>65 years old)†	0.03	(−0.07,0.01)	0.30	0.01	(−0.02,0.05)	0.37
% of urban population†	<0.01	(−0.01,0.01)	0.30	−0.01	(−0.01,0.01)	0.61
Gini (latest figure)	<0.01	(−0.01,0.01)	0.64	0.01	(−0.00,0.01)	0.31
Control of corruption	0.30^+^	(−0.01,0.61)	0.05	0.33*	(0.07,0.58)	0.01
Rule of law	−0.44	(−1.07,0.19)	0.17	−0.26	(−0.61,0.09)	0.15
Regulatory quality	−0.15	(−0.55,0.24)	0.44	0.30^+^	(−0.05,0.65)	0.15
Government effectiveness	0.16	(−0.38,0.70)	0.57	−0.38^+^	(−0.80,0.05)	0.08
Political stability and absence of violence/terrorism	0.06	(−0.17,0.28)	0.62	−0.06	(−0.22,0.09)	0.42
Voice and accountability	0.24^+^	(−0.02,0.50)	0.08	0.12	(−0.09,0.32)	0.27
Constant	−7.31**	(−8.97 to to 5.64)	<0.01	−3.14**	(−4.32 to to 1.95)	<0.01
Number of observations	102			102		
R-squared	0.96			0.95		

+ p<0.10, * p<0.05, ** p<0.01.

†All variables in percentages are coded in 0 to 100 number formats to ease their interpretation.

PHC, primary healthcare.

The first column shows the coefficients for government spending on PHC per capita. Significant predictors include government commitment to health, GDP per capita, tax revenue as % of GDP, control of corruption, and voice and accountability. Those significant predictors can be interpreted as follows:

- As suggested by the literature on determinants of total health spending, we found a significant effect of GDP per capita on the level of government spending on PHC. For example, when a country’s per capita GDP increases by 1% (eg, from US$ 100 to US$ 101 per capita), we would expect an increase in government spending for PHC by 1.01%, holding other variables constant. As the effect size is greater than 1, government spending on PHC is slightly income elastic. However, the effect on total PHC spending per capita is below 1, implying an inelastic relationship.
- Countries that allocate more public resources to health tend to have higher government expenditure on PHC, even after adjusting for GDP per capita. For example, if a government allocates an extra 1% of its general government spending to the health sector, we would expect to see a 7% increase in per capita government spending on PHC. We found no effect of higher government expenditure on health on total PHC spending as seen in column 3.
- We also found that countries which raise more money through taxation (including social contributions) have higher per capita government spending on PHC. For example, a 1 percentage point increase in the share of tax revenue in GDP is associated with a 4% increase in per capita government spending for PHC, holding other variables constant.
- Of all the political variables, two of the variables from the World Governance Index were found to be significantly associated with the level of government PHC expenditure, namely control of corruption and voice and accountability, although these are only significant at the 10% level. The positive signs on these variables can be interpreted to indicate that:
  - Governments with stronger control of corruption are more likely to spend more on PHC.
  - Governments in countries where their citizens are able to participate in selecting their government, and where there is freedom of expression and free media are more likely to spend more on PHC.

None of the social variables were significant predictors of government spending on PHC. As noted above, a number of social variables were excluded from the analysis due to missing values. When we included all of the social variables in the analysis, only the proportion of the elderly population and social capital were significant at 10% level (see appendix) but with negative signs for both of them and the estimation sample fell to 84.

The second column shows the coefficients for the predictors of total per capita PHC expenditure. As in the case of government spending on PHC, GDP per capita and tax revenue as a share of GDP were also significant predictors although with smaller coefficients. Government health spending as a share of general government spending was not associated with total PHC expenditure per capita, unlike in the case of public expenditure on PHC. Control of corruption was a strong predictor of total PHC spending per capita, with higher statistical significance compared with its effect on government spending on PHC. None of the social factors were shown significant even when we included all predictors into the model (see online supplemental appendix 2). We also re-ran the analysis by converting total PHC spending per capita, government spending on PHC per capita, and GDP per capita into international dollar (Purchasing Power Parity - PPP) (see online supplemental appendix 3). We did not detect any meaningful changes indicating the robustness of our analysis.

Table 5 shows the results of the multivariate regression of government spending on PHC as a share of total government health spending. The first column shows the results of the model excluding predictors that have missing values whereas the second column shows the model including all predictors which reduced the sample size from 102 to 84 countries.

**Table 5:** Results of the regression of the share of government spending on PHC (2019 or latest figure)

	Outcome: Government spending on PHC as a share of total government health spending					
Predictors	Effect size	95% CI	P value	Effect size	95% CI	P value
Government health spending as % of government spending in 2018†	−0.59	(−1.77 to 0.59)	0.32	−0.28	(−1.41 to 0.84)	0.62
Ln(GDP) per capita in 2018)	1.86	(−4.70 to 8.41)	0.57	2.84	(−4.62 to 10.31)	0.45
Tax revenue including social contribution as % of GDP (2018)†	0.02	(−0.54 to 0.58)	0.94	0.25	(−0.42 to 0.92)	0.46
% of working age population (15–65 years)†	0.12	(−0.48 to 0.72)	0.7	0.34	(−0.47 to 1.15)	0.4
% of >65 years†	−0.09	(−1.20 to 1.01)	0.87	−0.71	(−2.01 to 0.58)	0.28
% of urban population†	−0.07	(−0.29 to 0.14)	0.51	−0.16	(−0.40,0.09)	0.2
Gini (latest figure)	0.03	(−0.17 to 0.23)	0.76	−0.21	(−0.71 to 0.28)	0.4
Ethnic Fractionalisation Index (0–100)				0.04	(−0.12 to 0.20)	0.59
Population density (population per square kilometre area)				0	(−0.03 to 0.03)	0.91
Gender Inequality Index (0–100)				0.08	(−0.45 to 0.61)	0.75
Social Capital Index (0–100)				−0.37+	(−0.82 to 0.07)	0.1
Control of corruption	8.47*	(0.94 to 16.01)	0.03	7.09	(−5.79 to 19.97)	0.28
Rule of law	−12.07*	(-22.70 to 1.43)	0.03	−6.02	(−24.21 to 12.18)	0.51
Regulatory quality	−7.31	(−18.45 to 3.83)	0.2	−10.75	(−25.26 to 3.75)	0.14
Government effectiveness	5.81	(−6.96 to 18.59)	0.37	8.28	(−7.64 to 24.20)	0.3
Political stability and absence of violence/terrorism	−1.19	(−6.31 to 3.92)	0.64	−1.3	(−7.74 to 5.15)	0.69
Voice and accountability	7.37*	(1.50 to 13.24)	0.01	10.7	(−8.97 to 30.37)	0.28
POLITY regime measure				−0.01	(−1.40 to 1.39)	0.99
Government Closeness/Decentralisation Index				0.43	(−0.35 to 1.22)	0.27
Liberal Democracy Index				−0.13	(−0.64 to 0.37)	0.6
Constant	21.33	(−24.67 to 67.33)	0.36	29.97	(−64.52 to 124.46)	0.53
Number of observations	102			84		
R-squared	0.165			0.206		

*p<0.05, +p<0.1

†All variables in percentages are coded in 0 to 100 number formats to ease their interpretation.

PHC, primary healthcare.

None of the economic or social variables were statistically significant in either of the regressions, except for social capital which was significant in the all-predictor model. Its coefficient had a negative sign and was only significant at the 10% level, implying that higher social capital leads to a lower share of government spending on PHC. Given the more limited sample size, caution should be taken in interpreting this effect.

The positive signs on the coefficients of the World Governance Index can be interpreted as indicating that:

- Countries with stronger control of corruption are likely to allocate a higher share of public health funding to PHC.
- Countries with higher perceptions of effective judicial system and lower incidence of crime allocate a lower share of public health funding to PHC.
- Countries in which citizens are able to participate in selecting their government as well as freedom of expression and free media allocate a higher share of public funding to PHC.

Column 2 shows the results of the model with all predictors included. Comparing the two columns, control of corruption and rule of law are no longer significant.

We also conducted VIF analysis to explore the issue of multicollinearity among the predictors in our multiple regression model (see appendix 4). While multicollinearity does not reduce the explanatory power of the model, it might reduce the statistical significance of the predictors. There appears to be some multicollinearity between the variables of the World Governance Index. To some extent this is to be expected since they are all related to governance. Nevertheless, they measure distinct concepts and have therefore retained them in the model.

## Discussion

We examined economic, social and political factors associated with PHC expenditure using a cross section of countries for which data were available in the WHO Global Health Expenditure Database. Our analysis found that some economic factors—namely GDP per capita, government commitment to health and tax revenue raising capacity—were strongly associated with per capita government spending on PHC. We also found some evidence that the social and political factors for which we had data were associated with government spending on PHC per capita. Corruption was strongly associated with the level of total spending on PHC, while voice and accountability were positively associated with greater government commitment to PHC as measured by government spending on PHC as a share of total government health spending. Given the study design and limited comparable data, we consider the analysis as exploratory, able to provide preliminary insights on why some countries allocate more resources to PHC.

Consistent with the health expenditure literature,^21^ our results show that national income (measured as per capita GDP) is a strong determinant of PHC expenditure. Richer countries spend more money on PHC per capita, after controlling for other factors. Some authors argue that income is the only determinant of health expenditure that matters.^14 16 19^ As PHC expenditure is part of the total current health expenditure, it is expected that income would also show a positive association with PHC expenditure. However, this income effect may not extend to the share of PHC expenditure. Our findings provide no evidence that richer countries allocate more (or less) of the government health budget to PHC.

We found that a country’s capacity to raise tax revenue was positively associated with the level of government spending on PHC, holding income constant. Our measure of tax revenue combined both tax revenues and social contributions as evidence suggests that there is no difference in health expenditure between tax-based and insurance-based health financing arrangements.^21^ This implies that pooled funding, regardless of its financing arrangement, may help countries increase their government spending on PHC. Government allocation to health is also associated with higher level of government PHC spending. This finding aligns with a time series analysis of 195 countries showing governments’ increased prioritisation of the health sector as the strongest factor associated with increases in government health spending.^55^

We hypothesised that political factors would affect the level and share of government spending on PHC. Overall, we found that political factors may exert some influence on the level of spending and the prioritisation of PHC. Using the Polity Index, we found no evidence of the influence of the degree of democracy on either the level or share of government spending on PHC. This implies that a stronger policy for PHC is as likely to arise in a more democratic country as in an authoritarian regime. However, we found that control of corruption is strongly associated with the level of total spending on PHC, whereas voice and accountability is strongly associated with government spending on PHC as a share of total government health spending. We can only speculate as to the explanation for these findings as there is limited research on the role of political drivers on healthcare spending and governance. There are a number of possibilities—one is that governments that are (politically) invested in anticorruption agendas and improving access and equity, can mobilise larger share of resources for PHC against competing interests seeking to divert funds towards more lucrative areas such as hospitals services and technologies predominantly used by wealthier segments of the population sections.^56^ This may reflect strong political will, and also influence of effective bureaucracies or technocratic elites seeing benefits in PHC investment.^30^ Countries may also be better able to control corruption if they have stronger public financial management systems and can therefore execute and safeguard the health budgets efficiently.

The results on voice and accountability suggest that countries in which citizens have the means to engage with government can effectively advocate for greater prioritisation of PHC within the overall health budget. Nevertheless, emerging evidence suggests a more nuanced picture—citizen voice can promote financing for PHC primarily if enabled by organised political movements representing the interests and aspirations of grassroots constituencies.^13^ For example, in the case of Brazil broad-based social movements have been instrumental in the embedding right to health in the constitution, mobilising funding for PHC, directing it towards underserved populations and ensuring accountability through oversight by community-based and municipal structures.^13^

Other studies offer little insight as to whether political factors could influence PHC spending. One study in China and India found that development ideology and systematic pressure from the lower classes have had a major impact on government investment in public health,^34^ which is aligned with our finding that voice and accountability are associated with a greater share of government spending for PHC. A study of determinants of healthcare expenditure in 20 OECD countries found that the degree of territorial decentralisation tended to increase health expenditure.^25^ If the goal of the PHC approach is to bring health services closer to communities, it could be expected that a higher degree of decentralisation would be associated with a higher level or share of PHC spending but our study failed to provide evidence for this hypothesis.

The literature quantifying the role of political factors on PHC resource allocation is in its infancy. While our analysis provides some preliminary insights, better measures of political factors and stronger study designs that exploit longitudinal changes in the political context will be required to provide more rigorous evidence. Such an analysis is heavily data dependent and therefore may be most feasible by exploring within-country variation across subnational regions in countries with high quality data.

Our study has explored important determinants of PHC expenditure using a global data set that is comparable across countries. Other studies of PHC expenditure have tended to focus on one country or only on a specific aspect of PHC, such as primary care or pharmaceuticals for primary care. Turi *et al* analyse the determinants of outpatient expenditure within the primary care system in the city of Bauru, Brazil, and found that overweight, hypertension and physical inactivity were the main drivers of outpatient expenditure.^6^ In a different state of Brazil, Cabreira *et al* analysed PHC expenditure allocated to municipalities and argued that supply factors represented by the number of family health teams were stronger predictors than demand factors.^7^ In another setting, Kontopantelis *et al* investigated local variation in primary care funding in England.^8^ They found rurality and morbidity had the strongest positive association with funding, while weaker associations were found for deprivation and age structure. Finally, two studies analysed the determinants of pharmaceutical components of primary care expenditure and found that type of prescribed medicines (generic vs branded), and differences in prices were the main determinants in high-income countries,^9^ whereas Mujasi and Puig-Junoy found that number of outpatient visits, immunisation coverage, urbanisation and the number of government health facilities were the main predictors of drug expenditures in Uganda.^10^

### Limitations

There are several limitations to these analyses. First, use of the WHO’s broad PHC expenditure definition has the effect of biasing the estimates of PHC spending upwards because of the inclusion of outpatient services provided in hospitals. Any definition that uses a narrower scope would produce lower estimates of PHC expenditure. For example, the OECD reported that spending on primary care within ambulatory settings represented just 12% of current health expenditure; this increases to 17% when PHC services delivered in hospital settings are included, and to 34% by including retail pharmaceuticals.^57^ Agreeing to a common definition of PHC may be challenging as PHC involves a different set of services in each national setting, despite interest in having a consistent definition that can enable global comparisons.

Second, data are only available for a limited set of countries and for a single point in time. Most important for our purposes, the lack of consistent and comparable data on PHC expenditure over time means that it is not possible to easily identify which countries are increasing or sustaining their commitments to PHC. Estimates from small samples often exhibit sensitivity to small changes in the data set and the estimates reported here are no exception. Over time, more data on PHC spending will be available for more countries and be routinely available every year. A follow-up analysis is needed to test our hypotheses in a more robust manner, using methods such as panel data analysis. Regardless, we hope our study could be useful in providing some preliminary insight in how to increase prioritisation of financing PHC at the global level.

Third, we acknowledge that some political variables included in our model are not a perfect measure of governance and are probably highly correlated with each other, as indicated by the multicollinearity diagnosis test. We cannot be entirely confident about precisely which political factor is important, but our exploratory results suggest that there is merit in these political and governance factors that warrants further exploration. Given how these political variables are measured with error and change slowly, a further exploration to discern any specific relationship between financing PHC and political factors is better done in case studies.

## Conclusion

Many countries have committed to strengthen their PHC systems but it is unclear how this has been reflected in their health budgets. Monitoring PHC expenditure and understanding its determinants should allow a better understanding of how to prioritise PHC by increasing funding allocation towards it. Our analysis takes a step towards understanding of the drivers of PHC expenditure beyond the level of national income. Some of these drivers may be beyond the remit of health policy decision makers and relate to broader governance arrangements and political forces in societies. Thus, efforts to prioritise PHC in the health agenda and increase PHC expenditure require recognising the constraints within the political landscapes and engaging with a wide range of actors who influence decisions affecting the health sector.

## Data Availability

Data are available in a public, open access repository. All data are publicly available.

## Data availability statement

The data that support the findings of this study are available in public domain resources. All data are publicly available.
