# Supplementary material for: Determinants of government spending on primary healthcare: a global data analysis
Source: BMJ Glob Health. 2023 Nov 30;8(11):e012562. doi: 10.1136/bmjgh-2023-012562 (PMC10689394; doi:10.1136/bmjgh-2023-012562)
Supplement: Supplementary data [file bmjgh-2023-012562supp001.pdf]

Supplemental material for Determinants of government spending on primary health care: a global data analysis

Appendix 1 List of countries with available PHC expenditure data from WHO and OECD

No	Country	2016	2017	2018	2019	Latest	Included in the analysis?	Year if 2019 data is not available
1	Afghanistan	32.9	36.0	37.0	32.9	32.9	YES	
2	Armenia	168.8	193.7	201.2	249.7	249.7	YES	
3	Australia	1771.1	1862.7	1829.7	.	1829.7	YES	2018
4	Austria	1603.0	1680.6	1797.8	1762.1	1762.1	YES	
5	Belarus	.	.	131.9	152.5	152.5	YES	
6	Belgium	1784.8	1873.2	2007.2	1841.7	1841.7	YES	
7	Bhutan	40.4	43.1	42.2	53.7	53.7	YES	
8	Botswana	106.9	121.7	111.1	107.8	107.8	YES	
9	Burkina Faso	33.9	30.9	28.0	28.8	28.8	YES	
10	Cabo Verde Republic of	48.8	58.3	65.0	60.0	60.0	YES	
11	Cambodia	45.4	40.6	47.8	62.6	62.6	YES	
12	Canada	2097.4	2200.2	2265.0	2281.3	2281.3	YES	
13	Central African Republic	17.6	23.9	46.2	31.7	31.7	YES	
14	China	.	.	79.2	81.4	81.4	YES	
15	Colombia	208.1	229.6	.	.	229.6	YES	2017
16	Comoros	23.8	30.6	31.8	36.7	36.7	YES	
17	Congo	20.4	20.7	18.6	17.8	17.8	YES	
18	Costa Rica	314.6	205.5	178.5	193.5	193.5	YES	
19	Côte d'Ivoire	44.2	4.7	53.9	56.4	56.4	YES	
20	Czech Republic	438.2	482.8	554.5	558.4	558.4	YES	
21	Democratic Republic of the Congo	12.0	10.2	8.9	10.6	10.6	YES	
22	Denmark	2000.3	2089.1	2241.8	2197.7	2197.7	YES	
23	Dominican Republic	145.1	157.4	164.2	172.3	172.3	YES	
24	Egypt	66.3	.	55.1	.	55.1	YES	2018
25	Estonia	518.7	578.4	667.3	672.5	672.5	YES	
26	Eswatini	97.3	106.2	119.2	103.3	103.3	YES	
27	Ethiopia	17.1	16.9	16.6	18.6	18.6	YES	
28	Fiji	112.3	114.6	126.8	141.6	141.6	YES	
29	Finland	1893.9	1913.2	2032.1	2002.4	2002.4	YES	
30	Gabon	92.0	90.5	101.7	96.3	96.3	YES	
31	Georgia	134.9	.	.	.	134.9	YES	2016
32	Germany	2047.8	2209.3	2411.6	2419.2	2419.2	YES	
33	Ghana	48.8	49.3	55.1	.	55.1	YES	2018
34	Guinea	27.7	21.8	23.2	28.1	28.1	YES	
35	Guyana	165.4	.	215.0	232.9	232.9	YES	
36	Haiti	30.5	.	33.9	30.6	30.6	YES	
37	Hungary	371.3	386.0	413.0	419.7	419.7	YES	

38	Iceland	1729.3	2056.6	2177.8	2186.3	2186.3	YES	
39	India	25.1	.	24.8	26.7	26.7	YES	
40	Indonesia	.	.	.	27.9	27.9	YES	
41	Israel	548.3	607.7	.	.	607.7	YES	2017
42	Japan	2100.4	2079.7	2144.8	.	2144.8	YES	2018
43	Jordan	140.5	.	158.0	.	158.0	YES	2018
44	Kazakhstan	143.0	.	142.4	155.2	155.2	YES	
45	Kenya	40.7	37.8	42.2	45.5	45.5	YES	
46	Kyrgyz Republic	38.2	46.8	33.8	33.6	33.6	YES	
47	Lao People's Democratic Republic	32.4	36.7	30.0	32.2	32.2	YES	
48	Latvia	343.1	374.3	428.7	443.9	443.9	YES	
49	Liberia	41.4	30.3	27.8	26.7	26.7	YES	
50	Lithuania	470.9	516.8	581.7	653.5	653.5	YES	
51	Luxembourg	1883.0	1994.1	2193.2	1935.4	1935.4	YES	
52	Malawi	18.7	20.7	21.6	16.4	16.4	YES	
53	Malaysia	.	.	.	150.0	150.0	YES	
54	Mali	22.6	20.0	21.2	21.3	21.3	YES	
55	Mauritania	18.1	20.2	21.9	23.0	23.0	YES	
56	Mauritius	235.1	259.3	279.8	285.2	285.2	YES	
57	Montenegro	111.4	108.3	.	.	108.3	YES	2017
58	Morocco	.	.	82.7	82.3	82.3	YES	
59	Mozambique	17.9	20.9	23.7	.	23.7	YES	2018
60	Myanmar	37.8	37.4	38.4	38.7	38.7	YES	
61	Namibia	177.6	195.6	189.7	174.7	174.7	YES	
62	Nepal	29.0	30.3	29.6	30.1	30.1	YES	
63	Netherlands	1399.7	1455.2	1558.0	1541.1	1541.1	YES	
64	Niger	8.8	14.7	16.4	15.5	15.5	YES	
65	Nigeria	52.9	47.0	41.5	41.5	41.5	YES	
66	Norway	2768.4	2912.0	3068.0	2964.5	2964.5	YES	
67	Pakistan	20.2	.	21.2	19.2	19.2	YES	
68	Paraguay	.	134.6	156.1	126.5	126.5	YES	
69	Poland	366.9	406.4	446.9	427.6	427.6	YES	
70	Qatar	533.1	.	525.4	.	525.4	YES	2018
71	Republic of Korea	1099.3	1213.4	1366.3	1407.1	1407.1	YES	
72	Republic of Moldova	96.5	135.2	146.2	127.5	127.5	YES	
73	Saint Kitts and Nevis	645.5	.	599.1	.	599.1	YES	2018
74	Samoa	74.7	.	77.8	.	77.8	YES	2018
75	São Tomé and Principe	81.5	91.6	89.5	84.5	84.5	YES	
76	Senegal	33.3	33.6	38.8	36.5	36.5	YES	
77	Seychelles	285.8	379.7	408.2	410.1	410.1	YES	
78	Sierra Leone	.	23.6	21.6	22.9	22.9	YES	
79	Slovakia	526.9	535.0	573.9	580.7	580.7	YES	
80	Slovenia	724.3	772.3	871.1	895.1	895.1	YES	
81	South Africa	179.1	202.8	214.5	208.8	208.8	YES	
82	South Sudan	.	17.6	17.3	14.1	14.1	YES	
83	Spain	883.8	932.4	1009.9	986.9	986.9	YES	

Erlangga D, *et al. BMJ Glob Health* 2023; 8:e012562. doi: 10.1136/bmjgh-2023-012562

84	Sri Lanka	56.4	.	61.7	57.1	57.1	YES	
85	Suriname	149.0	.	197.1	248.2	248.2	YES	
86	Sweden	2094.5	2141.2	2205.9	2078.8	2078.8	YES	
87	Switzerland	3404.1	3494.0	3548.5	3520.2	3520.2	YES	
88	Tajikistan	26.0	26.2	26.8	27.0	27.0	YES	
89	Thailand	.	.	.	133.1	133.1	YES	
90	The Republic of North Macedonia	.	134.5	149.3	159.6	159.6	YES	
91	Timor-Leste	56.2	53.6	51.4	61.1	61.1	YES	
92	Togo	25.9	27.5	27.9	26.7	26.7	YES	
93	Tonga	67.2	.	.	.	67.2	YES	2016
94	Trinidad and Tobago	634.4	.	659.1	.	659.1	YES	2018
95	Tunisia	108.6	.	105.5	.	105.5	YES	2018
96	Uganda	21.7	16.9	17.5	17.7	17.7	YES	
97	United Arab Emirates	.	853.8	941.7	954.6	954.6	YES	
98	United Republic of Tanzania	11.9	13.0	13.8	13.0	13.0	YES	
99	Uzbekistan	56.8	41.9	34.1	57.8	57.8	YES	
100	Viet Nam	.	.	56.2	61.0	61.0	YES	
101	Zambia	39.9	46.3	52.9	47.8	47.8	YES	
102	Zimbabwe	.	48.9	61.0	44.0	44.0	YES	
103	Bosnia and Herzegovina	183.7	193.7	216.5	224.5	224.5	NO	
104	Brazil	291.3	340.9	308.2	297.1	297.1	NO	
105	Croatia	317.4	333.3	360.5	369.8	369.8	NO	
106	Cyprus	653.4	692.9	769.1	807.1	807.1	NO	
107	Iraq	.	.	.	139.6	139.6	NO	
108	Malta	1339.6	1496.1	1653.4	1528.4	1528.4	NO	
109	Mexico	200.6	204.9	209.1	219.3	219.3	NO	
110	Romania	181.3	186.2	221.1	250.1	250.1	NO	
111	Sudan	.	.	27.5	21.7	21.7	NO	
112	United Kingdom	1595.9	1556.7	1658.0	1650.9	1650.9	NO	
113	Uruguay	583.1	662.6	692.7	674.2	674.2	NO	

Appendix 2 Results of regression including all predictors (Outcome: total PHC spending and government spending on PHC)

	**Outcome: Government spending on PHC per capita**				**Outcome: Total PHC spending per capita**			
**Predictors**	**Effect**	**95% CI**	**Effect**	**95% CI**	**Effect**	**95% CI**	**Effect**	**95% CI**
Gov't health spending as % of gov't spending†	**0.07***	[0.01,0.13]	**0.08***	[0.02,0.13]	0.02	[-0.02,0.05]	0.01	[-0.02,0.05]
GDP per capita (USD)	**1.01****	[0.80,1.21]	**1.00****	[0.75,1.25]	**0.84****	[0.67,1.01]	**0.80****	[0.58,1.01]
Tax revenue including social contribution as % of GDP†	**0.04****	[0.01,0.06]	**0.06****	[0.02,0.09]	**0.02***	[0.00,0.04]	**0.02+**	[-0.00,0.04]
% of working age population (15-65 years)†	0.02	[-0.00,0.04]	0.03	[-0.01,0.06]	0	[-0.02,0.02]	0.01	[-0.02,0.03]
% of population > 65 years†	-0.01	[-0.05,0.03]	**-0.05+**	[-0.09,0.00]	0.01	[-0.02,0.05]	-0.01	[-0.05,0.03]
% Urban population†	0	[-0.01,0.00]	0	[-0.01,0.00]	0	[-0.01,0.00]	0	[-0.01,0.00]
Gini coefficient (latest figure)	0	[-0.01,0.01]	-0.01	[-0.02,0.01]	0	[-0.00,0.01]	0	[-0.02,0.02]
Control of corruption	**0.30+**	[-0.01,0.61]	0.12	[-0.24,0.49]	**0.33***	[0.07,0.58]	0.34	[-0.07,0.74]
Rule of law	-0.44	[-1.07,0.19]	0	[-0.51,0.50]	-0.26	[-0.61,0.09]	-0.22	[-0.70,0.25]
Regulatory quality	-0.15	[-0.55,0.24]	-0.18	[-0.64,0.29]	**0.30+**	[-0.05,0.65]	0.29	[-0.15,0.74]
Government effectiveness	0.16	[-0.38,0.70]	0.33	[-0.20,0.87]	**-0.38+**	[-0.80,0.05]	-0.38	[-0.90,0.14]
Political stability and absence of violence/terrorism	0.06	[-0.17,0.28]	0.01	[-0.25,0.27]	-0.06	[-0.22,0.09]	-0.08	[-0.28,0.13]
Voice and accountability	**0.24+**	[-0.02,0.50]	-0.07	[-0.69,0.55]	0.12	[-0.09,0.32]	0.3	[-0.35,0.95]
Ethnic fractionalisation index			0	[-0.00,0.01]			0	[-0.01,0.00]
Population density (population per sq km area)			0	[-0.00,0.00]			0	[-0.00,0.00]
Gender Inequality Index			0	[-0.02,0.03]			-0.01	[-0.02,0.01]
Social capital index			**-0.01+**	[-0.03,0.00]			-0.01	[-0.03,0.01]
POLITY regime measure			0.02	[-0.02,0.07]			-0.01	[-0.05,0.03]
Government closeness /decentralisation index			0.01	[-0.03,0.04]			0.02	[-0.01,0.04]
Liberal Democracy Index			0	[-0.02,0.02]			0	[-0.02,0.01]
Sample size	102		84		102		84	
R-squared	0.958		0.968		0.945		0.953	
BIC	189.8		175.1		151.3		154	

Appendix 3 Results of regression of PHC spending level data in international dollars (PPP)

	**Outcome: Government spending for PHC per capita (PPP)**				**Outcome: Total PHC spending per capita (PPP)**		
	Effect	95% CI	p-value		Effect	95% CI	p-value
**Gov't health spending as % of general gov't spending †**	**0.07***	**[0.01,0.13]**	**0.02**		**0.02**	**[-0.01,0.05]**	**0.28**
**Ln(GDP per capita)**	**1.06****	**[0.80,1.33]**	**0**		**0.69****	**[0.47,0.91]**	**0**
**Tax revenue including social contribution as % of GDP†**	**0.04****	**[0.01,0.06]**	**0.01**		**0.02***	**[0.00,0.04]**	**0.03**
**% of young people (<15 year)†**	**-0.02**	**[-0.04,0.01]**	**0.27**		**-0.01**	**[-0.03,0.01]**	**0.27**
**% of elderly > 65 year†**	**-0.03**	**[-0.07,0.02]**	**0.24**		**0.01**	**[-0.03,0.04]**	**0.78**
**% of Urban population†**	**0**	**[-0.01,0.00]**	**0.26**		**0**	**[-0.01,0.00]**	**0.8**
**Gini (latest figure)**	**0**	**[-0.01,0.01]**	**0.75**		**0**	**[-0.00,0.01]**	**0.44**
**Control of corruption**	**0.29+**	**[-0.02,0.60]**	**0.07**		**0.29***	**[0.04,0.53]**	**0.02**
**Rule of law**	**-0.46**	**[-1.10,0.17]**	**0.15**		**-0.28**	**[-0.66,0.09]**	**0.14**
**Regulatory quality**	**-0.21**	**[-0.61,0.19]**	**0.31**		**0.29**	**[-0.07,0.65]**	**0.11**
**Government effectiveness**	**0.2**	**[-0.34,0.73]**	**0.47**		**-0.28**	**[-0.73,0.18]**	**0.23**
**Political stability and absence of violence/terrorism**	**0.06**	**[-0.17,0.29]**	**0.6**		**-0.04**	**[-0.20,0.11]**	**0.56**
**Voice and accountability**	**0.26+**	**[-0.01,0.54]**	**0.06**		**0.11**	**[-0.11,0.33]**	**0.3**
**constant**	**1.41+**	**[-0.05,2.87]**	**0.06**		**3.65****	**[2.39,4.91]**	**0**
**Obs**	**102**				**102**		
**R-sqr**	**0.944**				**0.92**		
**BIC**	**191.4**				**148.3**		

Appendix 4 Variance Inflation Factor (VIF) analysis

Outcome 1: Government spending on PHC per capita		
**Variable**	**VIF**	**1/VIF**
**Government effectiveness**	**25.1**	**0.04**
**Rule of law**	**23.65**	**0.04**
**Regulatory quality**	**18.04**	**0.06**
**Ln(GDP per capita)**	**12.91**	**0.08**
**Control of corruption**	**11.07**	**0.09**
**% of elderly > 65 year†**	**7.82**	**0.13**
**% of young people (<15 year)†**	**6.75**	**0.15**
**Voice and accountability**	**4.69**	**0.21**
**Tax revenue including social contribution as % of GDP†**	**4.33**	**0.23**
**Political stability and absence of violence/terrorism**	**3.24**	**0.31**
**% of Urban population†**	**3.04**	**0.33**
**Gov't health spending as % of general gov't spending †**	**2.8**	**0.36**
**Gini (latest figure)**	**1.43**	**0.70**
**Mean VIF**	**9.61**	

Outcome 2: PHC Spending per capita		
**Variable**	**VIF**	**1/VIF**
**Government effectiveness**	25.1	0.04
**Rule of law**	23.65	0.04
**Regulatory quality**	18.04	0.06
**Ln(GDP per capita)**	12.91	0.08
**Control of corruption**	11.07	0.09
**% of elderly > 65 year†**	7.82	0.13
**% of young people (<15 year)†**	6.75	0.15
**Voice and accountability**	4.69	0.21
**Tax revenue including social contribution as % of GDP†**	4.33	0.23
**Political stability and absence of violence/terrorism**	3.24	0.31
**% of Urban population†**	3.04	0.33
**Gov't health spending as % of general gov't spending †**	2.8	0.36
**Gini (latest figure)**	1.43	0.70
Mean VIF	9.61	

Outcome 3: Share of government spending on PHC out of total government health spending		
**Variable**	**VIF**	**1/VIF**
**Government effectiveness**	**25.1**	**0.04**
**Rule of law**	**23.65**	**0.04**
**Regulatory quality**	**18.04**	**0.06**
**Ln(GDP per capita)**	**12.91**	**0.08**
**Control of corruption**	**11.07**	**0.09**
**% of elderly > 65 year†**	**5.64**	**0.18**

**% of young people (<15 year)†**	**4.69**	**0.21**
**Voice and accountability**	**4.33**	**0.23**
**Tax revenue including social contribution as % of GDP†**	**3.24**	**0.31**
**Political stability and absence of violence/terrorism**	**3.04**	**0.33**
**% of Urban population†**	**2.8**	**0.36**
**Gov't health spending as % of general gov't spending †**	**2.39**	**0.42**
**Gini (latest figure)**	**1.43**	**0.70**
**Mean VIF**	**9.1**	

Erlangga D, *et al. BMJ Glob Health* 2023; 8:e012562. doi: 10.1136/bmjgh-2023-012562
